# Supplementary material for: What does it take to learn the rules of RNA base pairing? A lot less than you may think
Source: bioRxiv. 2025 Aug 2:2025.07.31.668042. Preprint. [Version 1] doi: 10.1101/2025.07.31.668042 (PMC12324431; doi:10.1101/2025.07.31.668042)
Supplement: Supplement 1 [file media-1.gz › supplemental_material/src/rscape_v2.6.0.tar.gz!/rscape_v2.6.0/lib/infernal/Manuscripts/inf-1-appnote/changes.pdf]

BIOINFORMATICS APPLICATIONS NOTE

Vol. 00 no. 00 2008
Pages 1–3

Inferral 1.0: inference of RNA alignments

Eric P. Nawrocki,[1] Diana L. Kolbe[1] and Sean R. Eddy[1*]

[1]HHMI Janelia Farm Research Campus, Ashburn VA 20147, USA

Received on XXXXX; revised on XXXXX; accepted on XXXXX

Associate Editor: XXXXXXX

ABSTRACT

Summary: INFERNAL builds consensus RNA secondary structure profiles called covariance models (CMs), and uses them to search nucleic acid sequence databases for homologous RNAs, or to create new sequence- and structure-based multiple sequence alignments.

Availability: Source code, documentation, and benchmark downloadable from `http://infernal.janelia.org`. INFERNAL is freely licensed under the GNU GPLv3 and should be portable to any POSIX-compliant operating system, including Linux and Mac OS/X.

Contact: `{nawrockie,kolbed,eddys}@janelia.hhmi.org`

1 INTRODUCTION

When searching for homologous structural RNAs in sequence databases, it is desirable to score both primary sequence and secondary structure conservation. The most generally useful tools that integrate sequence and structure take as input any RNA (or RNA multiple alignment), and automatically construct an appropriate statistical scoring system that allows quantitative ranking of putative homologs in a sequence database (Gautheret and Lambert, 2001; Zhang *et al.*, 2005; Huang *et al.*, 2008). Stochastic context-free grammars (SCFGs) provide a natural statistical framework for combining sequence and (non-pseudoknotted) secondary structure conservation information in a single consistent scoring system (Sakakibara *et al.*, 1994; Eddy and Durbin, 1994; Brown, 2000; Durbin *et al.*, 1998).

Here, we announce the 1.0 release of INFERNAL, an implementation of a general SCFG-based approach for RNA database searches and multiple alignment. INFERNAL builds consensus RNA profiles called *covariance models* (CMs), a special case of SCFGs designed for modeling RNA consensus sequence and structure. It uses CMs to search nucleic acid sequence databases for homologous RNAs, or to create new sequence- and structure-based multiple sequence alignments. One use of INFERNAL is to annotate RNAs in genomes in conjunction with the RFAM database (Gardner *et al.*, 2009), which contains hundreds of RNA families. RFAM follows a seed profile strategy, in which a well-annotated "seed" alignment of each family is curated, and a CM built from that seed alignment is used to identify and align additional members of the family. INFERNAL has been in use since 2002, but 1.0 is the first version that we consider to be a reasonably complete production tool. It now includes E-value estimates for the statistical significance of database hits, and heuristic acceleration algorithms for both database searches and multiple alignment that allow INFERNAL to be deployed in a variety of real RNA analysis tasks with manageable (albeit high) computational requirements.

*to whom correspondence should be addressed

2 USAGE

A CM is built from a Stockholm format multiple sequence alignment (or single RNA sequence) with consensus secondary structure annotation marking which positions of the alignment are single stranded and which are base paired (Eddy, 2003). CMs assign position specific scores for the four possible residues at single stranded positions, the sixteen possible base pairs at paired positions, and for insertions and deletions. These scores are log-odds scores derived from the observed counts of residues, base pairs, insertions and deletions in the input alignment, combined with prior information derived from structural ribosomal RNA alignments. CM parameterization has been described in more detail elsewhere (Eddy and Durbin, 1994; Eddy, 2002; Klein and Eddy, 2003; Eddy, 2003; Nawrocki and Eddy, 2007).

INFERNAL is composed of several programs that are used in combination by following four basic steps:

1. Build a CM from a structural alignment with *cmbuild*.
2. Calibrate a CM for homology search with *cmcalibrate*.
3. Search databases for putative homologs with *cmsearch*.
4. Align putative homologs to a CM with *cmalign*.

The calibration step is optional and computationally expensive (4 hours on a 3.0 GHz Intel Xeon for a CM of a typical RNA family of length 100 nt), but is required to obtain E-values that estimate the statistical significance of hits in a database search. *cmcalibrate* will also determine appropriate HMM filter thresholds for accelerating searches without an appreciable loss of sensitivity. Each model only needs to be calibrated once.

3 PERFORMANCE

A published benchmark (independent of our lab) (Freyhult *et al.*, 2007) and our own internal benchmark used during development (Nawrocki and Eddy, 2007) both find that INFERNAL and other CM based methods are the most sensitive and specific tools for structural RNA homology search among those tested. Figure 1 shows updated results of our internal benchmark comparing INFERNAL 1.0 to the previous version (0.72) that was benchmarked in Freyhult *et al.* (2007), and also to family-pairwise-search with BLASTN

© Oxford University Press 2008.

(Altschul *et al.*, 1997; Grundy, 1998). INFERNAL's sensitivity and specificity have greatly improved, due mainly to three relevant improvements in the implementation (Eddy, 2003): a biased composition correction to the raw log-odds scores, the use of Inside log likelihood scores (the summed score of all possible alignments of the target sequence) in place of CYK scores (the single maximum likelihood alignment score), and the introduction of approximate E-value estimates for the scores.

The benchmark dataset used in Figure 1 includes query alignments and test sequences from 51 RFAM (release 7) families (details in (Nawrocki and Eddy, 2007)). No query sequence is more than 60% identical to a test sequence. The 450 total test sequences were embedded at random positions in a 10 Mb "pseudogenome". Previously we generated the pseudogenome sequence from a uniform residue frequency distribution (Nawrocki and Eddy, 2007). Because base composition biases in the target sequence database cause the most serious problems in separating significant CM hits from noise, we improved the realism of the benchmark by generating the pseudogenome sequence from a 15-state fully connected hidden Markov model (HMM) trained by Baum-Welch expectation maximization (Durbin *et al.*, 1998) on genome sequence data from a wide variety of species. Each of the 51 query alignments was used to build a CM and search the pseudogenome, a single list of all hits for all families were collected and ranked, and true and false hits were defined (as described in Nawrocki and Eddy (2007)), producing the ROC curves in Figure 1.

INFERNAL searches require a large amount of compute time (our 10 Mb benchmark search takes about 30 hours per model on average (Figure 1)). To alleviate this, INFERNAL 1.0 implements two rounds of filtering. When appropriate, the HMM filtering technique described by Weinberg and Ruzzo (2006) is applied first with filter thresholds configured by *cmcalibrate* (occasionally a model with little primary sequence conservation cannot be usefully accelerated by a primary sequence-based filter as explained in (Eddy, 2003)). The query-dependent banded (QDB) CYK maximum likelihood search algorithm is used as a second filter with relatively tight bands (β= 10^{-7}, the β parameter is the subtree length probability mass excluded by imposing the bands as explained in (Nawrocki and Eddy, 2007)). Any sequence fragments that survive the filters are searched a final time with the Inside algorithm (again using QDB, but with looser bands (β= 10^{-15})). In our benchmark, the default filters accelerate similarity search by about 30-fold overall, while sacrificing a small amount of sensitivity (Figure 1). This makes version 1.0 substantially faster than 0.72. BLAST is still orders of magnitude faster, but significantly less sensitive than INFERNAL. Further acceleration remains a major goal of INFERNAL development.

The computational cost of CM alignment with *cmalign* has been a limitation of previous versions of INFERNAL. Version 1.0 now uses a constrained dynamic programming approach first developed by Brown (2000) that uses sequence-specific bands derived from a first-pass HMM alignment. This technique offers a dramatic speedup relative to unconstrained alignment, especially for large RNAs such as small and large subunit (SSU and LSU) ribosomal RNAs, which can now be aligned in roughly 1 and 3 seconds per sequence, respectively, as opposed to 12 minutes and 3 hours in previous versions. This acceleration has facilitated the adoption of INFERNAL by RDP, one of the main ribosomal RNA databases (Cole *et al.*, 2009).

INFERNAL is now a faster and more sensitive tool for RNA sequence analysis. Version 1.0's heuristic acceleration techniques make some important applications possible on a single desktop computer in less than an hour, such as searching a prokaryotic genome for a particular RNA family, or aligning a few thousand SSU rRNA sequences. Nonetheless, INFERNAL remains computationally expensive, and many problems of interest require the use of a cluster. The most expensive programs (*cmcalibrate*, *cmsearch*, and *cmalign*) are implemented in coarse-grained parallel MPI versions which divide the workload into independent units, each of which is run on a separate processor.

ACKNOWLEDGEMENT

We thank Goran Ceric for his peerless skill in managing Janelia Farm's high performance computing resources.

Funding: INFERNAL development is supported by the Howard Hughes Medical Institute. It has been supported in the past by an NIH NHGRI training grant (T32-HG000045) to EPN, an NSF Graduate Fellowship to DLK, NIH R01-HG01363, and a generous endowment from Alvin Goldfarb.

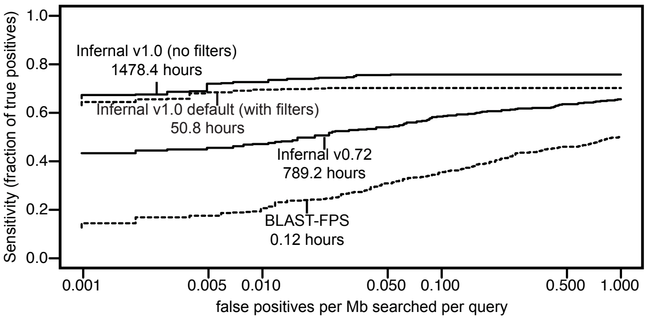

Fig. 1. ROC curves for the benchmark. Plots are shown for the new INFERNAL 1.0 with and without filters, for the old INFERNAL 0.72, and for family-pairwise-searches (FPS) with BLASTN. CPU times are total times for all 51 family searches measured for single execution threads on 3.0 GHz Intel Xeon processors. The INFERNAL 1.0 times do not include time required for model calibration.

REFERENCES

Altschul, S. F., Madden, T. L., Schaffer, A. A., Zhang, J., Zhang, Z., Miller, W., and Lipman, D. J. (1997). Gapped BLAST and PSI-BLAST: A new generation of protein database search programs. *Nucl. Acids Res.*, **25**, 3389–3402.

Brown, M. P. (2000). Small subunit ribosomal RNA modeling using stochastic context-free grammars. *Proc. Int. Conf. Intell. Syst. Mol. Biol.*, **8**, 57–66.

Cole, J. R., Wang, Q., Cardenas, E., Fish, J., Chai, B., Farris, R. J., Kulam-Syed-Mohideen, A. S., McGarrell, D. M., Marsh, T., Garrity, G. M., and Tiedje, J. M. (2009). The Ribosomal Database Project: Improved alignments and new tools for rRNA analysis. in press.

Durbin, R., Eddy, S. R., Krogh, A., and Mitchison, G. J. (1998). *Biological Sequence Analysis: Probabilistic Models of Proteins and Nucleic Acids*. Cambridge University Press, Cambridge UK.

Eddy, S. R. (2002). A memory-efficient dynamic programming algorithm for optimal alignment of a sequence to an RNA secondary structure. *BMC Bioinformatics*, **3**, 18.

Eddy, S. R. (2003). The Infernal user's guide. [http://infernal.janelia.org/].

Eddy, S. R. and Durbin, R. (1994). RNA sequence analysis using covariance models. *Nucl. Acids Res.*, **22**, 2079–2088.

Freyhult, E. K., Bollback, J. P., and Gardner, P. P. (2007). Exploring genomic dark matter: A critical assessment of the performance of homology search methods on noncoding RNA. *Genome Res.*, **17**, 117–125.

Gardner, P. P., Daub, J., Tate, J. G., Nawrocki, E. P., Kolbe, D. L., Lindgreen, S., Wilkinson, A. C., Finn, R. D., Griffiths-Jones, S., Eddy, S. R., and Bateman, A. (2009). Rfam: Updates to the RNA families database. NAR, in press.

Gautheret, D. and Lambert, A. (2001). Direct RNA motif definition and identification from multiple sequence alignments using secondary structure profiles. *J. Mol. Biol.*, **313**, 1003–1011.

Grundy, W. N. (1998). Homology detection via family pairwise search. *J. Comput. Biol.*, **5**, 479–491.

Huang, Z., Wu, Y., Robertson, J., Feng, L., Malmberg, R., and Cai, L. (2008). Fast and accurate search for non-coding rna pseudoknot structures in genomes. *Bioinformatics*, **24**, 2281–2287.

Klein, R. J. and Eddy, S. R. (2003). RSEARCH: finding homologs of single structured RNA sequences. *BMC Bioinformatics*, **4**, 44.

Nawrocki, E. P. and Eddy, S. R. (2007). Query-dependent banding (QDB) for faster RNA similarity searches. *PLoS Comput. Biol.*, **3**, e56.

Sakakibara, Y., Brown, M., Hughey, R., Mian, I. S., Sjölander, K., Underwood, R. C., and Haussler, D. (1994). Stochastic context-free grammars for tRNA modeling. *Nucl. Acids Res.*, **22**, 5112–5120.

Weinberg, Z. and Ruzzo, W. L. (2006). Sequence-based heuristics for faster annotation of non-coding RNA families. *Bioinformatics*, **22**, 35–39.

Zhang, S., Haas, B., Eskin, E., and Bafna, V. (2005). Searching genomes for noncoding RNA using FastR. *IEEE/ACM Trans. Comput. Biol. Bioinform.*, **2**, 366–379.
